# SARS-CoV-2 Prevalence in a Delivering Refugee Population: Refugee Status, Payor Type, Race, and Vaccination Status

**DOI:** 10.1007/s10903-024-01645-y

**Published:** 2024-12-06

**Authors:** Eleanor U. Johnston, Bikash Bhattarai, Crista E. Johnson-Agbakwu, Dean V. Coonrod

**Affiliations:** 1 Creighton University School of Medicine Phoenix Regional Campus, Phoenix, AZ USA; 2 Department of Obstetrics, Gynecology & Women’s Health, Valleywise Health/District Medical Group, Phoenix, AZ USA; 3 University of Arizona College of Medicine-Phoenix, Phoenix, AZ USA

**Keywords:** COVID-19, Health disparities, Labor and delivery, Public safety net, Refugees, Universal testing

## Abstract

Underserved communities were disproportionately affected during the coronavirus (COVID-19) pandemic. Limited data exist on the impact of COVID-19 among refugee populations because refugee status is not often classified in electronic medical record (EMR) systems, unlike race or primary language. The study aim was to evaluate the PCR-based prevalence of SARS-CoV-2 in a delivering population over the first 2 years of the pandemic by refugee status, ethnicity, insurance, and vaccination status. A cross-sectional study examined parturient patients admitted to an urban safety-net hospital from May 2020 to May 2022 who were tested for SARS-CoV-2on admission. Percentages and prevalence ratios of SARS-CoV-2 between refugee status, insurance type, vaccination status, and race/ethnicity were calculated across four time periods, corresponding with variant surges of the pandemic. 3,502 patients delivered, 476 (13.6%) were refugees. Self-pay (46.4%) and Medicaid (46.4%) were the most frequent insurance types with a Hispanic predominance (64.5%) by race/ethnicity. Only 12.8% of patients received at least one vaccine before delivery: 13.2% in non-refugees versus 10.3% refugees 192 (5.5%) of the mothers tested positive during the study period with 6.1% refugees positive versus 5.4% among non-refugees, (prevalence ratio, 1.13; *P* = 0.53, 95% confidence interval [0.77, 1.66]). Positive tests ranged between 4.7% and 6.3% across insurance types and between 4.4% and 7.5% across race/ethnicity categories. The highest prevalence ratio (refugee/non-refugee) of 2.01 was during the Delta Surge (*P* = 0.12, 95% confidence interval [0.84, 4.82]) and the lowest prevalence ratio of 0.64 was during the Omicron Surge (*P* = 0.21, 95% CI [0.32, 1.30]). Among refugees when examined by primary language, 51.7% of positive tests were from those speaking languages of the African Great Lakes region (Kinyarwanda, Kirundi, Swahili, Kiswahili). We observed only small differences in SARS-CoV-2 prevalence between refugees and non-refugees or in vaccination status. Variations in prevalence ratio were seen by refugee status by variant surge. Subsets of the refugee population, when grouped by language/region, appeared to be more affected. This warrants future research on the impact of the SARS-CoV-2 pandemic on specific refugee communities, rather than refugee communities as a heterogenous unit.

## Background

Refugees, along with ethnic minorities and immigrants, faced higher rates of infection, hospitalization, and mortality throughout the pandemic [1, 2]. Despite this gap, refugee status is often not classified in electronic medical record (EMR) systems, unlike race or primary language. EMR data on refugee populations could improve knowledge of health trends in these communities and, accordingly, contribute to policy implementation that better serves these patients.

In 2020, a research study conducted at Valleywise Health Medical Center (VHMC), a public safety net hospital in Phoenix, Arizona, determined patient refugee status via primary language spoken, country of nativity, and affiliation with a local refugee resettlement agency [3]. The study found that SARS-CoV-2 disproportionately affected refugee populations at the onset of the pandemic, from May to July 2020. This study expands that original dataset by 22 months, from May 2020 to May 2022. The goal of this study is to understand SARS-CoV-2 trends across the four stages of the pandemic and to compare the test positivity in refugees and non-refugee labor and delivery patients.

## Theoretical/Conceptual Framework

This study, like its predecessor at VHMC, implements sentinel surveillance as a powerful public health tool for monitoring progression of SARS-CoV-2. Sentinel surveillance identifies a defined condition (e.g., SARS-CoV-2) in a population sample and uses the in-sample data to indicate trends in a larger target population [4]. Labor and delivery patients provide an apt sample for sentinel surveillance because they present from all socioeconomic classes and life circumstances [5]. Universal testing of all labor and delivery patients also captures asymptomatic cases. This is essential in an infection like COVID-19, with asymptomatic rates between one-sixth to one-fifth of all cases [6, 7].

By following prevalence ratios (PR) of SARS-CoV-2 in delivering refugee populations at VHMC over the first two years of the pandemic, the current study aims to investigate whether the disproportionate effect of COVID-19 on refugee populations continued, and if specific groups of refugees—when stratified by primary language—were affected more than others. Public health interventions such as education and accessible vaccines and testing can then be improved by targeting these populations.

## Methods

### Participants

A retrospective cohort study was performed on all patients who were admitted to the labor and delivery unit at VHMC from May 7, 2020, to May 5, 2022, using the hospital’s EMR.

### Data Collection

At the time of their labor and delivery admission, each patient was tested for SARS-CoV-2 via a nasal pharyngeal swab with the rapid Cepheid Xpert Spress SARS-CoV-2 polymerase (PCR) assay. During the time frame of this study, VHMC was enforcing a universal testing policy for labor and delivery unit admittees. Under the universal testing policy, all patients admitted to labor and delivery were tested for SARS CoV-2. Patients who refused testing or who left against medical advice before testing could be completed were excluded from the final dataset.

Non-eligible patients were removed from the dataset. This included those with a prior positive COVID test in the 90 days before admission for delivery and those with no test 5 days before or after delivery. Patients with a positive test within 90 days prior to admission were excluded because a positive test at time of admission would have been impossible to distinguish between prior or current infection [8]. Patients who delivered prior to 20 weeks or did not give birth were also excluded as these were considered separate conditions and thus might consist of a heterogenous group of subjects and not representative of a sentinel surveillance cohort. Deduplication of multiple records of the same patient due to multiple births or other reasons was also done by matching medical record number and admission date.

### Measures

Refugee status was determined using an internal refugee registry. This registry was based on the following criteria pulled from the EPIC database: language, country of origin, refugee health examination (ICD-10 code) on problem list, refugee patient type (a designation used in the women’s clinic), and refugee visit types scheduled.

Once refugee status was determined, prevalence of COVID-19 was compared over time in refugee versus non-refugee parturient patients. Refugee status was the primary independent variable. Other patient demographics and known COVID-19 risk factors were also abstracted from the dataset including race, ethnicity, language spoken at home, gravidity, parity, insurance status, and vaccination status. Asymptomatic versus symptomatic infection was determined by manual chart review of SARS-COV-2 positive patients. The patient was considered symptomatic if any symptoms were noted in the medical record (fever, cough, rhinorrhea, pharyngitis, anosmia, myalgias). Patients were only classified asymptomatic if no symptoms were recorded, including on a standard intake nursing checklist, and/or if infection was labeled “asymptomatic” in patients’ medical record.

### Analysis

Overall PCR-based prevalence of SARS-CoV-2 was calculated for all patients eligible for the study. To evaluate overall change in proportion of patients testing positive to SARS-CoV-2 PCR, prevalence was evaluated across four major time periods based on surge dates (Table 1). A Cochran-Armitage trend test was used to evaluate difference in PCR-positive percentages across these four periods.

**Table 1:** Four time periods of analysis used in this study

Period Title	Dates^a^
Period 1: Original Surge	05/07/2020–12/31/2020
Period 2: Second Wave	1/01/2021–03/20/2021
Period 3: Delta Surge	03/21/2021–08/30/2021
Period 4: Omicron Surge	08/31/2021–5/05/2022

^a^These four time periods were determined for their correspondence with variant surges of the COVID-19 pandemic, from May 2020 to May 2022 [9]

Prevalence across specific demographic and clinically distinct groups was analyzed to evaluate the association between the factor of interest and PCR-positive proportions. Prevalence ratios and 95% confidence limits for patients with and without the attributes of interest were presented and compared to identify patient-factors attributable to higher prevalence. Since refugee status was our factor of primary interest, the analyses were stratified and presented by refugee status.

This study was approved by the Valleywise Health Institutional Review Board (IRB protocol number 2020-044).

## Results

From May 7th, 2020, to May 5th, 2022, there were 3,502 patients who delivered that were eligible for the study. Of the 3,502 patients, 476 (13.6%) were refugees. Self-pay (46.4%) and Medicaid (43.6%) were the most frequent insurance types with Hispanic predominance (64.5%) by race/ethnicity. Only 12.8% of patients had received at least one vaccine before delivery: 13.1% in non-refugees versus 10.3% refugees.

When comparing the refugee with the nonrefugee population (Table 2), Medicaid and Self-pay were the most frequent insurance types across both groups. The refugee population had a Black or African American (58.6%) predominance by race/ethnicity whereas the nonrefugee population had a Hispanic or Latino predominance (74.1%) by race/ethnicity. Notably, although refugees made up only 13.6% of overall patients, they represented more than half of the Black or African American patients (61.0%) delivering at VHMC within the study timeframe.

**Table 2:** Demographic breakdown of eligible patients by refugee versus nonrefugee

Patient Characteristics	Overall	Refugee	Nonrefugee
Total	3502	476	3026
**Race and Ethnicity**	**n (%)**	**n (%)**	**n (%)**
Black or African American	457 (13.05)	279 (58.61)	178 (5.88)
Hispanic or Latino	2258 (64.48)	17 (3.57)	2241 (74.06)
Mixed and/or all others	492 (14.05)	103 (21.64)	389 (12.86)
White, non-Hispanic	295 (8.42)	77 (16.18)	218 (7.20)
**Insurance Type**	**n (%)**	**n (%)**	**n (%)**
Medicaid	1528 (43.63)	308 (64.71)	1220 (40.32)
Self-pay	1625 (46.40)	116 (24.37)	1509 (49.87)
Private	304 (8.68)	51 (10.71)	253 (8.36)
Other	45 (1.28)	1 (0.21)	44 (1.45)
**COVID-19 Vaccination**	**n (%)**	**n (%)**	**n (%)**
≥ 1 Vaccination	448 (12.80)	49 (10.29)	399 (13.19)
No vaccination	3054 (87.20)	427 (89.71)	2627 (86.81)

Regarding SARS-Cov-2 detection, 192 (5.5%) of the mothers tested positive during the two-year study period with 6.1% refugees positive versus 5.4% among non-refugees, PR 1.13 (*P* = 0.53, 95% CI [0.77, 1.66]), as seen in Table 3. Positive tests ranged between 4.7% and 6.3% across the insurance types and between 4.4% and 7.5% across race/ethnicity categories. Of all parturient patients testing positive for COVID, most cases (78.1%) were asymptomatic.

**Table 3:** Overall prevalence of positive tests by refugee status from May 2020 to May 2022

	Detected *n* (%)	Not detected *n* (%)	Total	Prevalence Ratio (95% CI)
Refugee	29 (6.09)	447 (91.6)	476	1.13 (0.77–1.66)
Non-refugee	163 (5.39)	2863 (93.65)	3026	

**Fig. 1:**
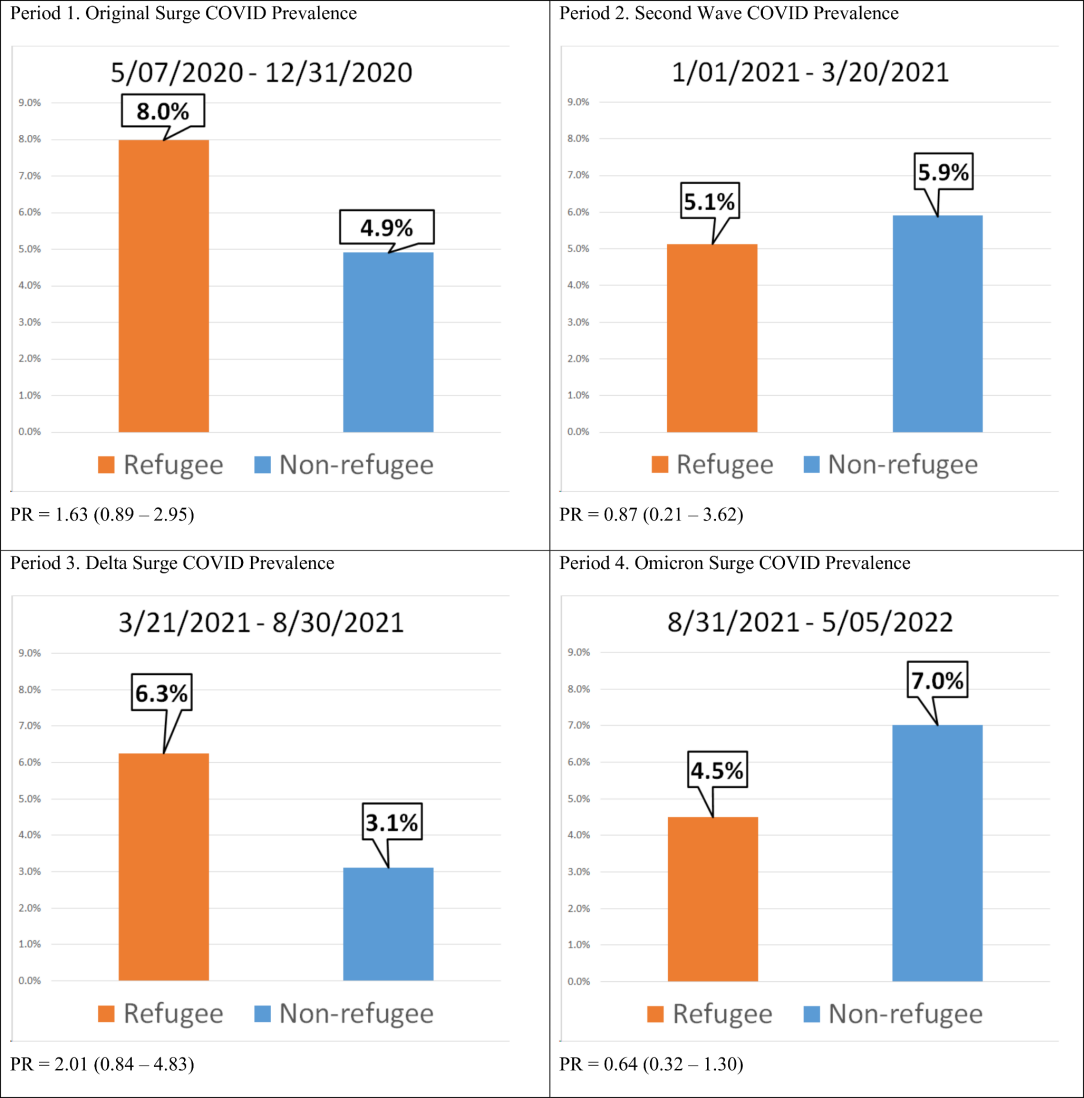
SARS-CoV-2 prevalence by refugee status across four time periods, corresponding with variant surges of the pandemic, from May 2020 to May 2022. Prevalence of COVID-19 in refugees versus non-refugees for each period is displayed as prevalence ratio (PR) with corresponding *P*-value. There was no statistically significant difference (*P* < 0.05) between SARS-CoV-2 prevalence in refugees versus non-refugees across the four periods. *PR*, Prevalence Ratio

The 2-year period was further divided into 4 stages, corresponding with variant surges of the pandemic, to better visualize changes in prevalence over time. PCR positivity across the four periods among non-refugees ranged from 3.11 to 7.018 (Cochran-Amitrage trend test *P* = 0.01). Test positivity in refugees ranged from 4.49 to 7.98 (*P* = 0.65) and 3.49 to 6.68 for all patients (*P* = 0.02). The highest PR (refugee/non-refugee) of 2.0 (*P* = 0.12) was during the Delta Surge period and lowest PR of 0.6 (*P* = 0.21) during the Omicron Surge period (Fig. 1).

**Fig. 2:**
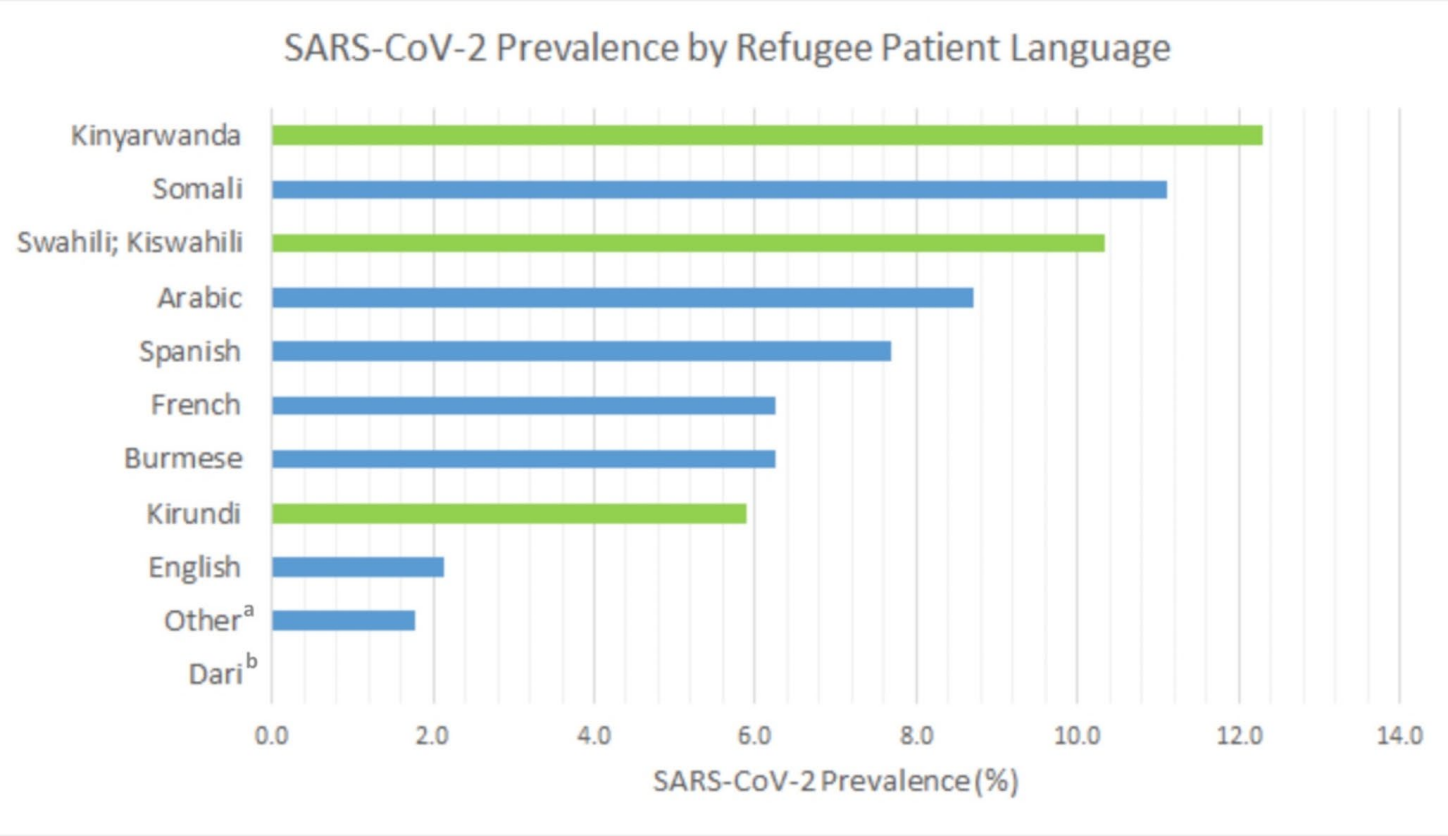
SARS-CoV-2 prevalence by primary language listed for refugee patient. Only languages with greater than a 10-patient sample are listed. Languages spoken in the African Great Lakes region are highlighted in green, including Kinyarwanda, Swahili, and Kirundi. These patient populations had the first, third, and eighth highest SARS-CoV-2 prevalence, respectively. ^a^Languages with less than a 10-patient sample were combined into the “Other” category. ^b^Zero out of 20 Dari-speaking refugees tested positive for SARS-CoV-2

Among the refugee group, over 29 different languages were listed as primary language. The most frequent primary languages listed were English (29.2%), Kinyarwanda (13.6%), Swahili/Kiswahili (12.2%), and Arabic (9.7%). The remaining 168 refugee patients spoke over 25 languages. Among the most frequent primary language groups, the populations with the highest SARS-CoV-2 prevalence were those speaking Kinyarwanda (12.31%), Somali (11.11%), and Swahili/Kiswahili (10.34%), as seen in Fig. 2. Kinyarwanda and Swahili are languages of the African Great Lakes region, and Somali is spoken in countries of the Great Lakes region as well as in Somalia [10, 11]. Of note, the refugee patient group that listed English as their primary language was the largest group of refugees (29.2%) but had among the lowest SARS-CoV-2 prevalence (2.2%). The lowest SARS-CoV-2 prevalence (0.0%), among primary language groups with a sample size greater than 10 patients, was seen in Dari-speaking refugees (Fig. 2).

## Discussion

We observed only small differences in SARS-CoV-2 prevalence between refugees (6.1%) and non-refugees (5.4%) or in vaccination status. This contrasts with the earlier study conducted at the same urban safety net hospital at the very onset of the pandemic. That study found that COVID-19 disproportionately affected refugee populations, with a prevalence of 17.8% for refugee patients and 8.2% for non-refugee patients (prevalence ratio, 2.16; 95% confidence interval, 1.04–4.51) [3].

Subsets of the refugee population when grouped by language/region appeared to be more affected by SARS-CoV-2. When examined by primary language, the refugee populations with the highest SARS-CoV-2 prevalence were those speaking Kinyarwanda (12.31%), Somali (11.11%), and Swahili/Kiswahili (10.34%). Kinyarwanda and Swahili are languages of the African Great Lakes region, which Somalia is adjacent to [10, 11]. On the other hand, the group of refugee patients that listed English as their primary language compromised the largest group of refugees but had among the lowest SARS-CoV-2 prevalence (2.2%).

The disappearance of a significant difference in prevalence between refugee and non-refugee populations as the pandemic progressed may support a hypothesis that populations infected at higher levels earlier in the pandemic were protected in later periods due to immunity. Other possible explanations include improved social distancing policies and pandemic protocols for frontline workers, increased understanding of infectivity and quarantine guidelines, and increased availability of multilingual or culturally competent medical information about the pandemic. In fact, in response to our early findings we found that contact tracing was only conducted in English and Spanish, so we partnered with public health authorities to expand contact tracing language capabilities [3].

Cultural health navigators (CHN) employed by the Refugee Women’s Health Clinic at the hospital may also have contributed to the disappearance of higher COVID prevalence in the hospital’s refugee population as the pandemic progressed. CHNs are multicultural, multilingual individuals who serve communities of which they are also members, either having resettled themselves as refugees from the same regions and/or by sharing socio-cultural, linguistic, racial/ethnic, and religious commonalities [12, 13]. CHNs served as essential frontline workers for the refugee community at the hospital during the pandemic—they were trusted messengers who facilitated linkages to care, dispelled myths, and provided social support. Their work may have led to higher penetration of COVID-19 mitigation efforts over time.

An investigation of primary language and health impact would be incomplete without an acknowledgement of varying literacy rates and English language proficiency across the refugee populations examined in our study. Refugees generally have limited literacy and English proficiency [14]. In one 2023 qualitative study of refugee women in Arizona, it was noted that some literate refugee women used online translating apps to translate text messages from their clinics, but the illiterate were unable to do so, and overall, most refugee patients could not communicate in English [14]. During the COVID-19 pandemic, VHMC sought to combat these language disparities by releasing educational videos on COVID-19 vaccines in eleven languages. The videos were released from 2020 to 2021 and included Burmese, Kirundi, Lingala, Maay Maay, Spanish, Arabic, Somali, S’gaw Karen, Kinyarwanda, and Swahili translations [15]. These videos sought to increase vaccine awareness, reduce misconceptions, and reduce hesitancy to receive vaccines. Together they have more than 200,000 views, with the Somali video alone reaching 87,000 viewers [15]. The timely release of these videos, which feature the aforementioned Cultural Health Navigators, may also have contributed to the disappearance of higher COVID prevalence in the hospital’s refugee population. However, when considering electronic resources, it is important to take accessibility into account. Immigrants in Arizona are disproportionately likely to lack access to the internet or a computer/laptop compared to their native-born counterparts [16].

The variance in SARS-CoV-2 prevalence seen in subsets of the refugee population serves as a potent reminder that heterogeneity exists within disadvantaged populations. SARS-CoV-2 prevalence was highest in refugee patients listing their primary language as Kinyarwanda (12.31%), Somali (11.11%), or Swahili/Kiswahili (10.34%) and lowest in those listing English as their primary language (2.2%). One explanation is that refugees in the three groups with the highest SARS-CoV-2 prevalence were living within the same communities in Phoenix and transmitting the same cases among each other. The refugee group listing English as their primary language, on the other hand, was a larger, more diverse group of refugees and thus may have been less vulnerable to single-community viral spread or may have been better linked to messages and services for English-speaking populations. Nevertheless, this is a powerful reminder that language barriers are inextricably linked to health outcomes, as shown in several prior studies [17, 18]. The variance in SARS-CoV-2 prevalence across refugee groups warrants future research on the impact of the SARS-CoV-2 pandemic on specific refugee groups, rather than refugees as a single heterogenous unit. This approach is not unique to SARS-CoV-2 and is seen in infectious disease analysis of other migrant populations, including Chagas disease in Latin Americans and human immunodeficiency virus (HIV) in Sub-Saharan Africans [19].

## New Contribution to the Literature

This study implements sentinel surveillance as a powerful public health tool for monitoring progression of SARS-CoV-2. Sentinel surveillance identifies a defined condition (e.g., SARS-CoV-2) in a population sample and uses the data to indicate trends in a larger target population [4]. Labor and delivery patients provide an apt sample for sentinel surveillance because they present from all socioeconomic classes and life circumstances, independent of virus-related symptoms [5]. One shortcoming of this patient population, however, is that it is entirely composed of females of childbearing age. Thus, generalizability of findings is limited. Nevertheless, data from sentinel surveillance studies—specifically those involving universal testing at public safety net hospitals with a focus on the marginalized groups they predominantly serve—can be used to inform public health interventions.

This study also emphasizes the necessity of identifying refugee populations in electronic medical record systems. Previous research has declared this a critical step in meeting the healthcare needs of this population and has shown promise in using machine-learning methods to meet this important goal [20]. Electronic medical record systems themselves have both strengths and weaknesses as a primary source for data collection. While simple and efficient for analyzing data, EMRs may over-simplify information as data entry is often limited to pre-defined categories (e.g. preset race, ethnicity, language options) that may not capture nuances in individual patients. Additionally, data entry in EMRs is susceptible to human error and operator subjectivity which may skew analysis. For example, determination of asymptomatic versus symptomatic status in this study was reliant on individual charting of patient symptoms and thus susceptible to operator subjectivity.

One strength of this study was that universal testing of patients admitted to the labor and delivery unit captured high rates of asymptomatic SARS-CoV-2 cases. Unlike other areas of the hospital where patient admission is dependent on display of pathologic symptoms, the labor and delivery unit provides a unique location for measuring asymptomatic rates of diseases in the community [5]. In this study, 78% of positive patients were asymptomatic cases. This correlates to the findings of similar studies conducted via universal testing of obstetric patients, with most studies reporting that over 66% of patients were asymptomatic at the time of testing [21, 22].

It is no secret that the COVID-19 pandemic disproportionately affected populations of color [3, 23]. Public safety net hospitals have played and continue to play an integral role in addressing that disparity. Among communities of color, refugee populations—and specifically refugee women—are especially vulnerable [24]. Phoenix is a major refugee destination in the southwest United States. The Refugee Women’s Health Center at VHMC alone has served over 9000 refugees from over 60 countries across sub-Saharan Africa, Southeast Asia, and the Middle East [3, 25]. Despite the prevalence and vulnerability of these refugee populations, it is often difficult to abstract refugee data from electronic medical record systems. This study and the refugee registry at VHMC seek to change that pattern and increase inclusion of refugee populations in public health research [20].

## Declarations

### Paper Presentation Information

The XXIV FIGO World Congress of Gynecology and Obstetrics, International Federation of Gynecology and Obstetrics (FIGO), Paris, France. October 9–12, 2023.
